# Prediction of frequency response of sub-frame bushing and study of high-order fractional derivative viscoelastic model

**DOI:** 10.1038/s41598-024-66536-6

**Published:** 2024-07-09

**Authors:** Bao Chen, Lunyang Chen, Feng Zhou, Jiang Huang, Zehao Huang

**Affiliations:** 1 Key Laboratory of Advanced Manufacturing Technology for Automobile Parts, Ministry of Education, Chongqing University of Technology, Chongqing, 400054 China; 2 School of Vehicle Engineering, Chongqing University of Technology, Chongqing, 400054 China

**Keywords:** Sub-frame bushing, BP neural network, High-order fractional derivative new model, Dynamic stiffness, Parameter identification, Engineering, Mechanical engineering

## Abstract

This paper presents experimental and dynamic modeling research on the rubber bushings of the rear sub-frame. The Particle Swarm Optimization algorithm was utilized to optimize a Backpropagation (BP) neural network, which was separately trained and tested across two frequency ranges: 1–40 Hz and 41–50 Hz, using wideband frequency sweep dynamic stiffness test data. The testing errors at amplitudes of 0.2 mm, 0.3 mm, and 0.5 mm were found to be 1.03%, 3.05%, and 1.96%, respectively. Subsequently, the trained neural network was employed to predict data within the frequency range of 51–70 Hz. To incorporate the predicted data into simulation software, a dynamic model of the rubber bushing was established, encompassing elastic, friction, and viscoelastic elements. Additionally, a novel model, integrating high-order fractional derivatives, was proposed based on the frequency-dependent model for the viscoelastic element. An enhanced Particle Swarm Optimization algorithm was introduced to identify the model's parameters using the predicted data. In comparison to the frequency-dependent model, the new model exhibited lower fitting errors at various amplitudes, with reductions of 3.84%, 3.61%, and 5.49%, respectively. This research establishes a solid foundation for subsequent vehicle dynamic modeling and simulation.

## Introduction

Rubber bushings are widely used in automotive chassis, especially in suspension components. They provide a flexible connection between two interconnected parts, reducing wear and improving the lifespan of the components. In many electric vehicle chassis, replacing hinged connections with rubber bushings can enhance components durability. Additionally, rubber bushings offer advantages of lower cost and lighter weight compared to hinges, contributing to reduce manufacturing costs and overall vehicle weight.

Rubber bushings play a crucial role in vehicles by connecting the suspension to the body through subframe bushings, providing support for multidirectional loads^1^. The transmission of road forces and shocks to the body can be reduce, thereby improving overall NVH (noise, vibration, and harshness) performance of the vehicle^2^. Rubber exhibits strong nonlinear viscoelastic properties within the bushing, which are significantly influenced by factors such as load amplitude, frequency, and operating cycles. Therefore, the development of accurate rubber bushing models holds great significance in improving suspension and vehicle dynamic simulation precision^3,4^.

Extensive research has been conducted by scholars worldwide on the dynamics of rubber bushings, with early studies primarily focusing on linear models such as the Kelvin–Voigt, the Zener and the Maxwell model^5^. Chinese scholar Beibei Sun^6^ introduced the concept of the rubber bushing as a combination of elastic, viscoelastic, and frictional elements, which provided a clearer understanding of rubber bushing dynamic modeling. Given the strong nonlinear viscoelastic properties of the rubber material within the bushing, research on the viscoelastic elements of the bushing model has become a focal point.

The most common dynamic models for viscoelastic elements in rubber bushings are standard mechanical models such as the Kelvin-Voigt, Maxwell, Dzierzek^7^, and Frequency-dependent model^8^. To better represent the viscoelasticity of rubber, fractional derivative models have been proposed. Metzler, Bagley, Nonnenmacher, Liu and Lin Song, have respectively employed fractional derivative models to study the viscoelastic properties of rubber bushings^9–12^. A five-parameter fractional derivative model is proposed by Zhao^13^.

In simulation software such as MSC Adams, rubber bushing is expressed in the form of a dynamic model, so a more accurate rubber bushing dynamic model can help improve the accuracy of the simulation model in the simulation software. Further improve the reliability of pre-product development and product optimization.

**Figure 1:**
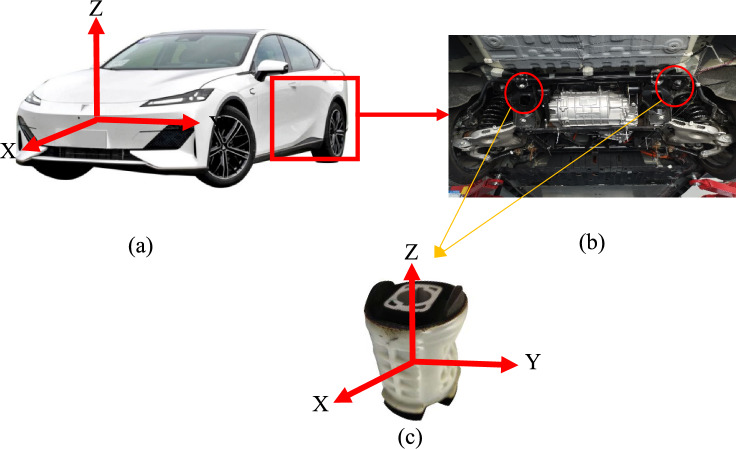
(**a**) is an actual vehicle; (**b**) is an actual vehicle chassis; (**c**) is the rubber bushing form the actual vehicle.

This paper focuses on the rubber bushing of the rear subframe of a vehicle. The rubber bushing is shown in Fig. 1, where the X-direction represents the radial solid direction of the rubber bushing, the Y-direction represents the radial hollow direction of the rubber bushing, and the Z-direction represents the axial direction of the rubber bushing. The stiffness of different directions is different, so this paper focuses on Y-direction.

The rubber bushing was subjected to experimental analysis, with a focus on the Y-direction. The PSO-BP (Particle Swarm Optimization-Backpropagation) neural network was trained and tested across two frequency ranges: 1–40 Hz and 41–50 Hz. The trained PSO-BP neural network was then used to predict data in the frequency range of 51–70 Hz. In addition, to improve the accuracy of the rubber bushing's dynamic model, a high-order fractional derivative new model was proposed based on the Frequency-dependent model. The new model aimed to enhance the overall model accuracy. Then parameter identification was performed on the dynamic model, and a modified particle swarm optimization algorithm was proposed for parameter identification.

## Prediction of the dynamic characteristics of rubber bushing

### Rubber bushing experiment

**Figure 2:**
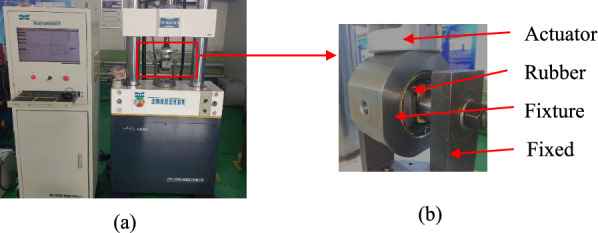
(**a**) is the LETRY dynamic stiffness testing platform; (**b**) is the rubber bushing X loading.

Experiments are the most effective and intuitive method for studying the mechanical properties of rubber bushings. In this study, the rear-point rubber bushing of a vehicle rear suspension sub-frame was selected as the experimental object. Dynamic and static loading tests were conducted on the rubber bushing to obtain experimental data. The LETRY dynamic stiffness testing platform, as shown in Fig. 2, was used for the experiments.

#### Rubber bushing static loading test

The elastic and friction units of the bushing model established in this study are used to simulate the static behavior of the bushing.

**Figure 3:**
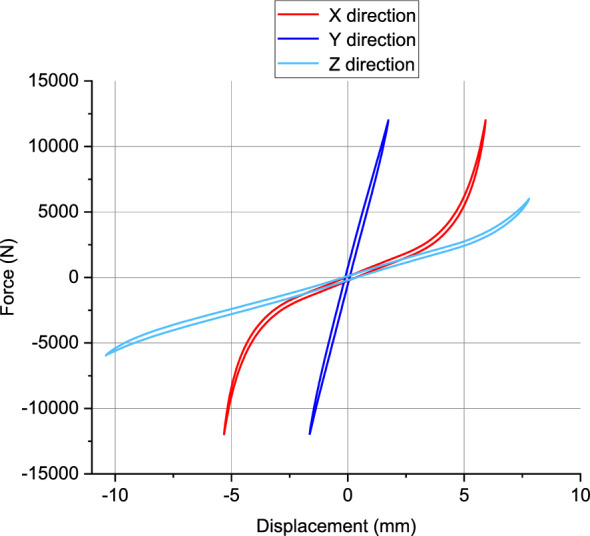
Rubber bushing static loading test data in X/Y/Z-directions.

The loading range in the X/Y direction is ± 12000N, and in the Z direction is ± 6000N. The experimental results are shown in Fig. 3. Due to the anisotropic nature of the rubber bushing studied in this paper, the static characteristics in the X/Y/Z directions exhibit significant differences.

#### Rubber bushing dynamic loading test

The dynamic loading test of the rubber bushing involves conducting wideband frequency sweep tests with sinusoidal excitations of different amplitudes. To fully investigate the dynamic characteristics of the rubber bushing, dynamic loading tests were performed in the frequency range of 1–50 Hz with amplitudes of 0.2 mm, 0.3 mm, and 0.5 mm. The relationship curve between the dynamic stiffness of the rubber bushing and the sweep frequency was obtained.

**Figure 4:**
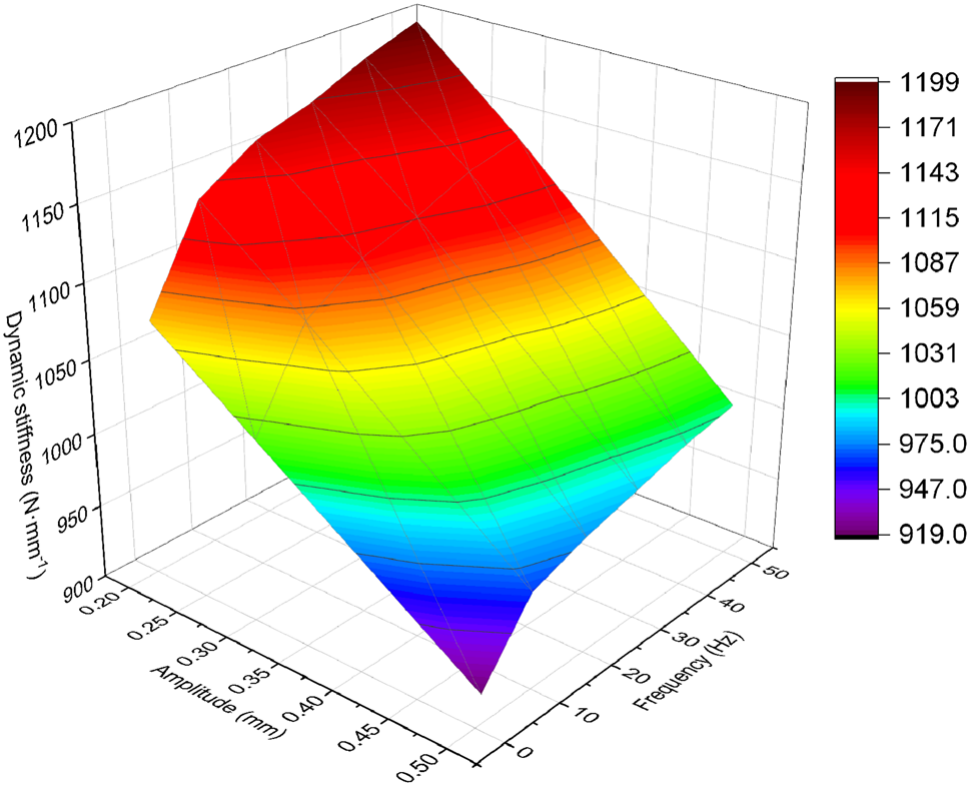
Relationship between dynamic stiffness and frequency in X-direction for different amplitudes of rubber bushing.

**Figure 5:**
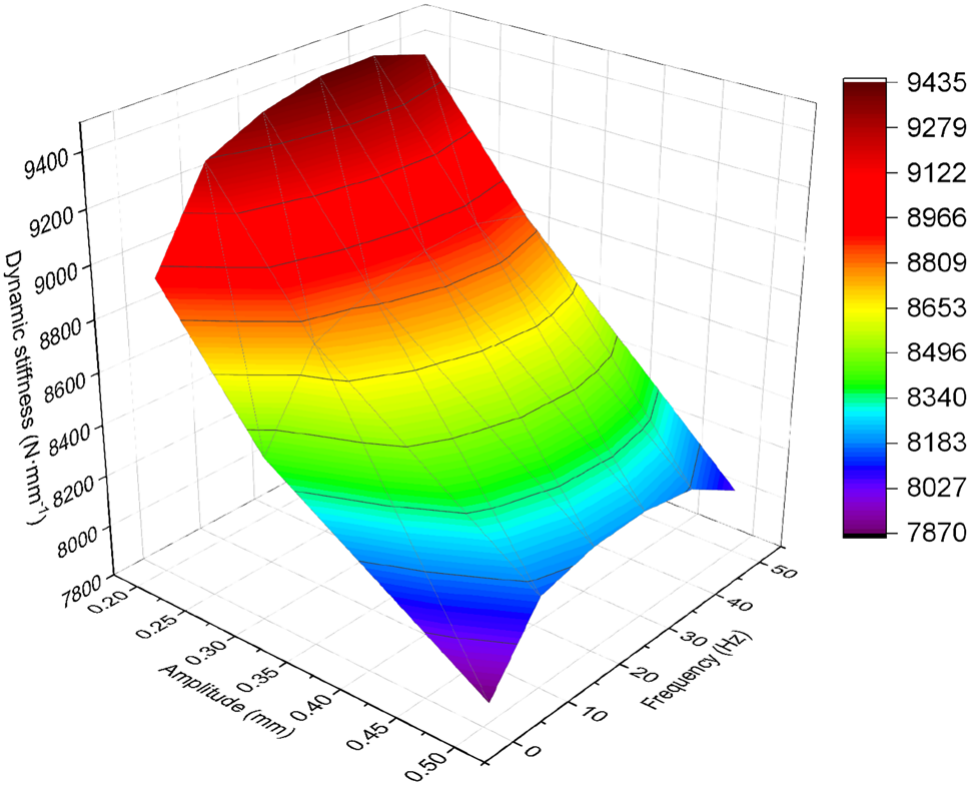
Relationship between dynamic stiffness and frequency in Y-direction for different amplitudes of rubber bushing.

**Figure 6:**
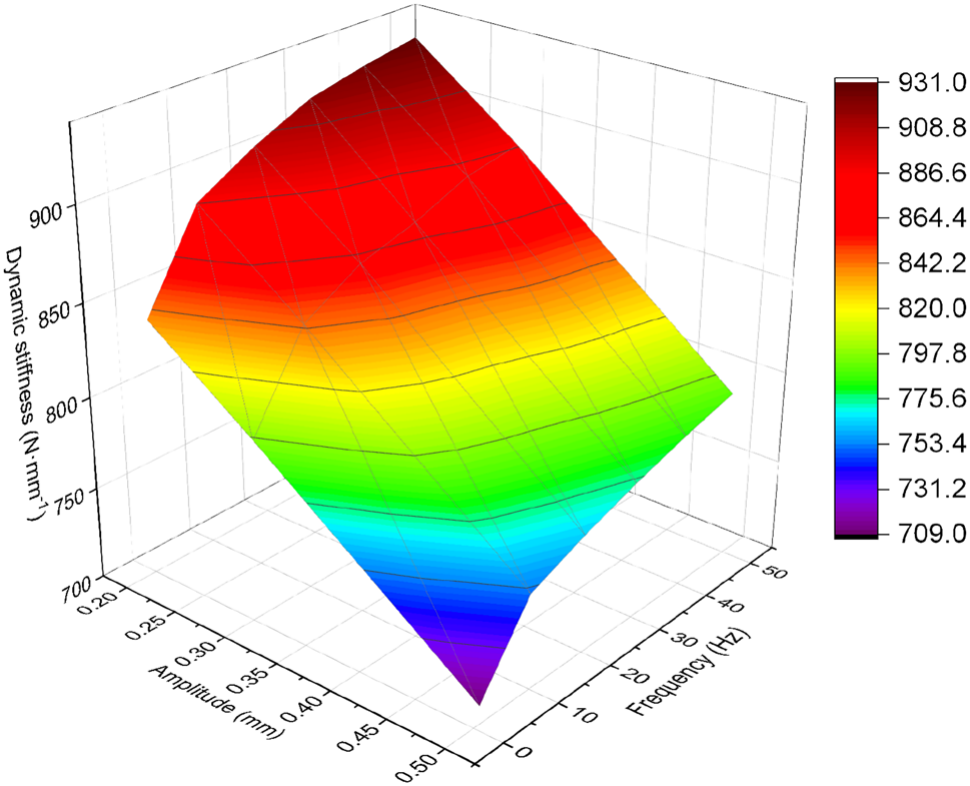
Relationship between dynamic stiffness and frequency in Z-direction for different amplitudes of rubber bushing.

The experimental results for the X, Y, and Z directions are shown in Figs. 4, 5, 6.

In the case of constant amplitude, the dynamic stiffness of the rubber bushing in the X/Y/Z directions increases with increasing frequency. Conversely, under constant frequency, the dynamic stiffness decreases with increasing amplitude. There is significant variation in the dynamic stiffness of the rubber bushing in different directions and amplitudes, particularly noticeable between the X and Y directions. In this study, the focus was on modeling the rubber bushing in the Y-direction, which exhibits higher dynamic stiffness.

### Prediction of Dynamic Stiffness in the Y-Direction of Rubber Bushing using PSO-BP Neural Network

In the testing of rubber bushings, wideband frequency sweep tests are required at different amplitudes. This is especially crucial in the research process of NVH characteristics, where more frequency test samples are needed. However, the increased demand for test samples leads to higher costs and longer testing cycles. Additionally, during the testing process, the resonance of the testing machine itself can cause abrupt changes in the test data of the rubber bushings^14^.

To reduce the testing cost and cycle, BP neural networks are used to predict the test data of rubber bushings. In order to improve the prediction accuracy of the BP neural network, this study combines it with the PSO algorithm^15^. The BP neural network adjusts its network weights and thresholds based on the prediction error^16^. The PSO algorithm, proposed by Dr. Eberhart and Dr. Kennedy in 1995^17^, is an intelligent optimization algorithm inspired by birds searching for food. It searches for the optimal solution based on fitness by updating the position and velocity of particles.

The iteration formulas for updating velocity and position in the PSO algorithm is calculated as follows:

1 $$ \begin{gathered} v_{t + 1} = wv_{t} + c_{1} r_{1} (p_{b} - x_{t} ) + c_{2} r_{2} (g_{b} - x_{t} ) \hfill \\ x_{t + 1} = x_{t} + v_{t + 1} \hfill \\ \end{gathered} $$

In Eq. 1, $w$ represents the inertia weight; ${v}_{t}$ and ${x}_{t}$ denote the current particle's velocity and position; ${p}_{b}$ and ${g}_{b}$ respectively indicate the positions associated with the individual best fitness value and the global best fitness value; ${r}_{1}$ and ${r}_{2}$ represent random numbers within the range (0,1); and ${c}_{1}$ and ${c}_{2}$ are the learning factors; the velocity and position of the particle have ranges of $[{v}_{min},{v}_{max} ]$ and $[{x}_{min},{x}_{max} ]$, respectively.

The fitness function for the PSO-BP neural network can be expressed as follows:

2 $$ F = k\left( {\sum\limits_{i = 1}^{n} {\left| {(y_{i} - o_{i} )} \right|} } \right) $$

In the formula, $n$ represents the number of output nodes in the neural network, ${y}_{i}$ denotes the desired output of the i-th node in the BP neural network, and ${o}_{i}$ represents the predicted output of the i-th node. The coefficient $k$ is set to 1 in this study.

**Figure 7:**
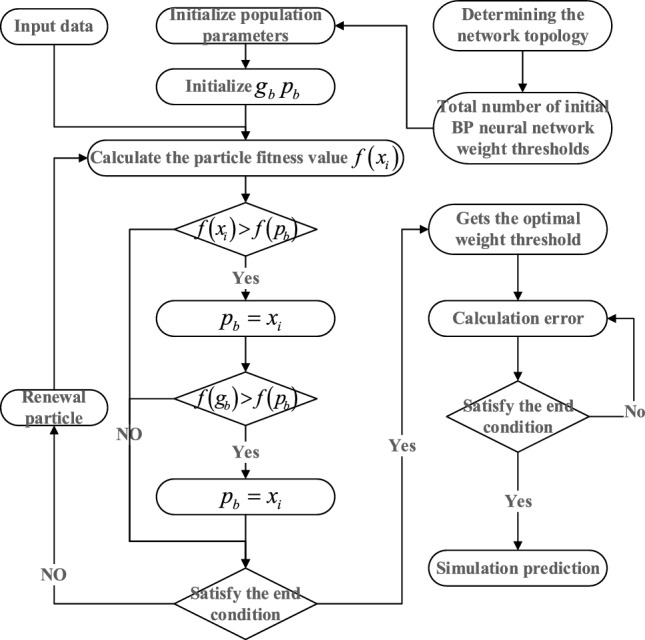
PSO-BP neural network flowchart.

The parameter settings are provided in Table 1, and the PSO-BP neural network process is illustrated in Fig. 7.

**Table 1:** Parameter Settings for PSO-BP Neural Network.

Algorithm	Parameters	Values
BP neural network	Input neuron	1
	Hidden layer	5
	Output neuron	1
	Training samples	1–40 Hz
	Test samples	41–50 Hz
	Prediction	51–70HZ
	Learning rate	0.1
PSO algorithm	Particle dimension	16
	Population size	30
	Number of iterations	50
	$$[{v}_{min} , {v}_{max}]$$	[− 1,1]
	$$[{x}_{min} , {x}_{max}]$$	[− 5,5]
	${c}_{1}$	1.5
	${c}_{2}$	1.5

The particle dimension refers to the sum of the threshold and weight count of the entire neural network. The weight count is calculated as follows: 1 × 5 + 5 × 1 = 10, and the threshold count is 5 + 1 = 6. Therefore, the particle dimension is 10 + 6 = 16.

**Figure 8:**
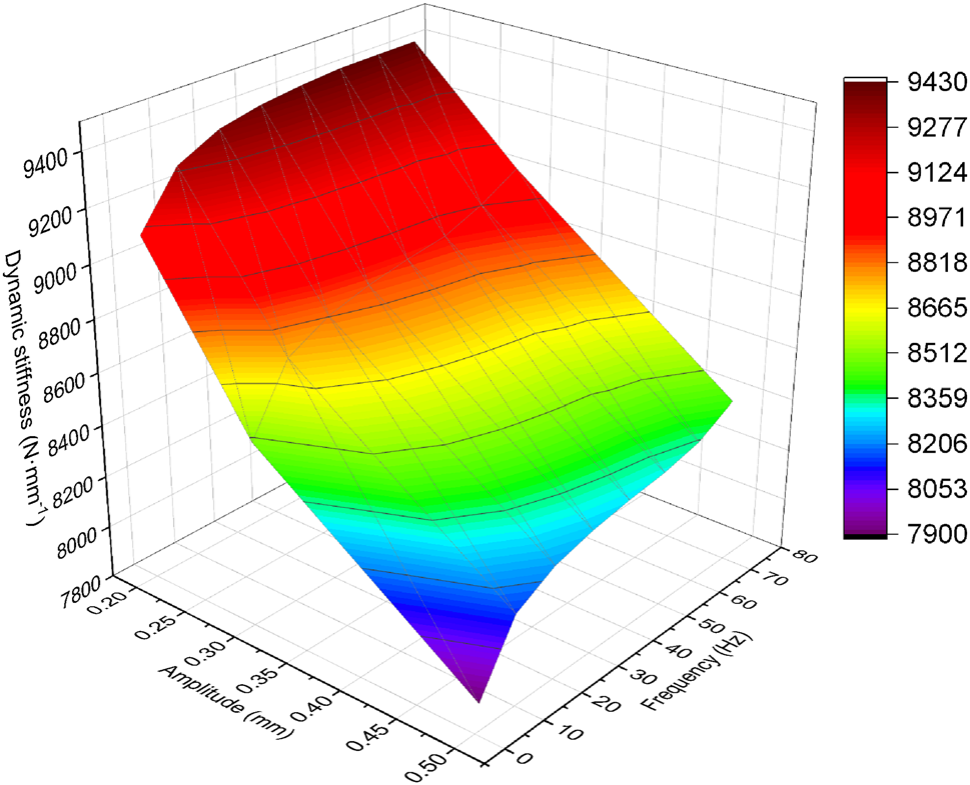
1–70 Hz, prediction results of PSO-BP neural network.

**Figure 9:**
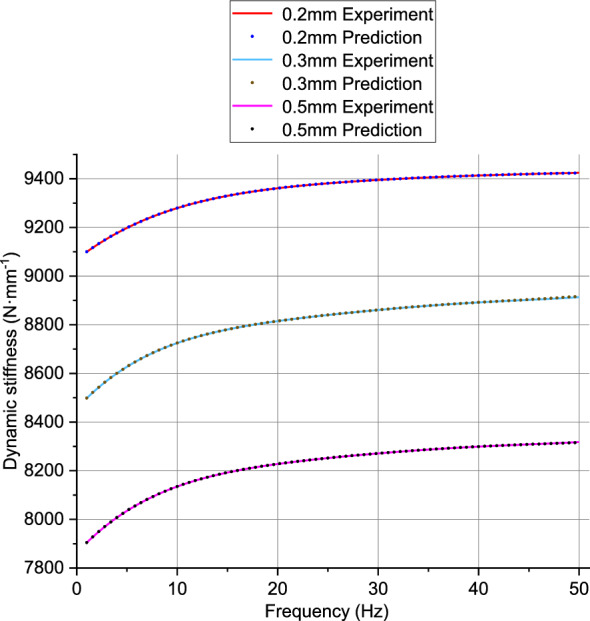
Comparison of prediction results from PSO-BP neural network with experimental data.

The prediction results based on PSO-BP neural network test data can be shown by Fig. 8. According to Fig. 9, the comparison between the prediction results and experimental data results of the PSO-BP neural network can be observed.

**Figure 10:**
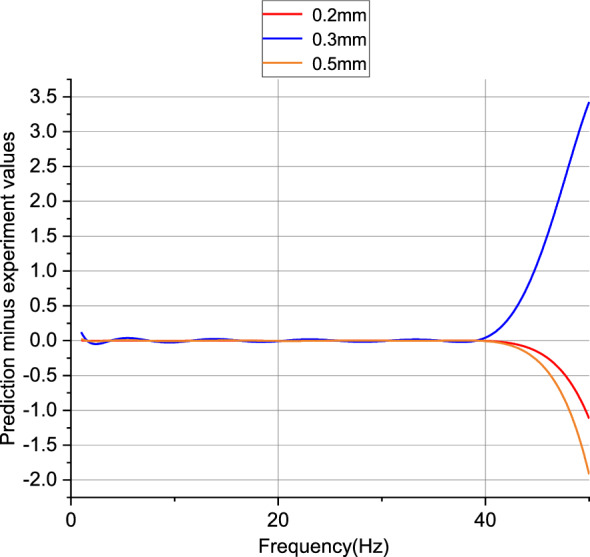
Difference between predicted values and experimental values for 1–50 Hz.

According to Fig. 10, the data samples ranging from 1 to 40 Hz are used as training data, resulting in lower prediction errors. The samples ranging from 41 to 50 Hz are used to test the capabilities of the trained PSO-BP neural network, hence the errors may be higher compared to the 1–40 Hz range. Table 2 shows the calculated errors of the neural network, and errors within 10% are considered acceptable. From the table, it can be seen that the prediction errors are within an acceptable range.

**Table 2:** Prediction errors of the PSO-BP neural network.

Amplitude	Percentage Error (%)
0.2 mm	1.03
0.3 mm	3.05
0.5 mm	1.96

## Establishment of the parameterized model for rubber bushing.

**Figure 11:**
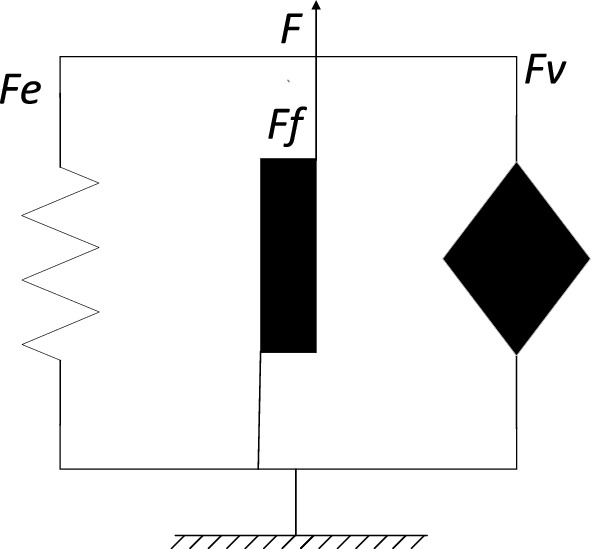
Rubber bushing dynamic model.

The dynamic model of the bushing, as shown in Fig. 11, can be constructed by parallel connections of elastic, frictional, and viscoelastic elements. In the figure, ${F}_{e}$ represents the elastic force in units of $\text{N}$; ${F}_{f}$ represents the force of the frictional hysteresis element in units of $\text{N}$;${F}_{v}$ and represents the viscoelastic force in units of $\text{N}$; $F$ represents the response force of the entire parameterized model in units of $\text{N}$.

Since the elastic, frictional, and viscoelastic elements are connected in parallel, the combined force of the three elements represents the response force of the entire bushing, as expressed in Eq. 3:

3 $$ F = F_{e} + F_{f} + F_{v} $$

### Elastic element

The static characteristics of the bushing are caused by its elastic deformation. Constitutive models commonly used to describe the static mechanical behavior include the Mooney-Rivlin model^18^, Neo-Hookean model^19^, Yeoh model^20^, Ogden model^21^, etc. However, considering the elastic deformation characteristics of the bushing, in order to more flexibly accommodate the nonlinearity of the elastic element, a polynomial spring model is used to represent the static characteristics of the bushing. The polynomial spring can adjust the highest degree or coefficients to adapt to the nonlinearity of the elastic element. Its mechanical expression is as follows^22^:

4 $$ F_{e} = a_{0} + a_{1} x + a_{{_{2} }} x^{2} + ...... + a_{n} x^{n} $$

${F}_{e}$ represents the force of the elastic element, measured in units of $\text{N}$. Under the influence of a sinusoidal excitation with an amplitude of ${x}_{0}$, the amplitude of the elastic module is given by:

5 $$ F_{e0} = a_{0} + a_{1} x_{0} + a_{{_{2} }} x_{0}^{2} + ...... + a_{n} x_{0}^{n} $$

The elastic element does not consider friction, so there is no energy loss.

### Frictional element

The hysteresis effect of a rubber bushing becomes more pronounced with increasing deformation, and the nonlinearity becomes more evident. The expression for the smooth friction force model is as follows^23^:

6 $$ F_{f} = F_{fs} + \frac{{\left( {x - x_{s} } \right)\left[ {F_{f\max } - sign\left( {\dot{x}} \right)F_{fs} } \right]}}{{x_{2} \left[ {1 - sign\left( x \right)\frac{{F_{fs} }}{{F_{f\max } }}} \right] + sign\left( {\dot{x}} \right)\left( {x - x_{s} } \right)}} $$

Among them, ${F}_{f}$ represents the frictional force, x represents the displacement of the loading, measured in units of mm; ${F}_{fmax}$ is the maximum frictional force, measured in units of N; ${x}_{2}$ is the displacement at which the frictional force increases from 0 to ${F}_{fmax}/2$, measured in units of mm;(${x}_{s}$, ${F}_{fs}$) represents a reference point on the force–displacement curve obtained from static loading tests. Under the influence of a sinusoidal excitation with an amplitude of ${x}_{0}$, the amplitude of the frictional hysteresis module is given by:

7 $$ F_{f} = \frac{{F_{f\max } }}{{2x_{2} }}\left( {\sqrt {x_{0}^{2} + x_{2}^{2} + 6x_{0} x_{2} } - x_{0} - x_{2} } \right) $$

8 $$ E_{f} = 2F_{f\max } \left[ {2x_{0} - x_{2} \left( {1 + u} \right)^{2} \ln \frac{{x_{2} \left( {1 + u} \right) + 2x_{0} }}{{x_{2} \left( {1 + u} \right)}}} \right] $$

In the formula,$u= {F}_{f0}/{F}_{fmax}$, and ${E}_{f}$ represents the energy dissipation per cycle, measured in units of N∙mm.

### Viscoelastic element

For the dynamic model of the viscoelastic element in a rubber bushing, following the approach proposed by Liu Guo jia et al., a high-order fractional derivative model is derived based on the Frequency-dependent model.

The Frequency-dependent model is then modified to develop a new model called the High-Order Fractional Derivative Frequency-dependent model. The structure of the Frequency-dependent model and the new model is shown in Table 3.

**Table 3:** Model Structure Diagram.

Model	Structure
Frequency dependent	
New model	

#### Frequency-dependent model

The mechanical expression of the Frequency-dependent model is as follows:

9 $$ F_{1} = k_{1} x $$

10 $$ F_{2} = k_{2} z + c_{2} \dot{z} = c_{1} \left( {\dot{x} - \dot{z}} \right) $$

11 $$ F = F_{1} + F_{2} $$

In the equations:$x$ represents the loading displacement of the rubber bushing, measured in units of mm; z represents the displacement of the elastic element ${k}_{2}$ and the damping element ${c}_{2}$, measured in units of mm; $k_{1} ,k_{2} ,c_{1} ,c_{2}$ are the elastic coefficients and damping coefficients of the model.

Substituting Eqs. 9 and 10 into Eq. 11:

12 $$ F = k_{1} x + c_{1} \left( {\dot{x} - \dot{z}} \right) $$

In the formula, $\dot{z}$ represents:

13 $$ \dot{z} = \frac{1}{1 + \beta }\left( {\dot{x} - \frac{\alpha }{\gamma }z} \right) $$

$$\alpha = \frac{{k}_{2}}{{k}_{1}}$$; $$\beta = \frac{{c}_{2}}{{c}_{1}}$$ ; $$\gamma = \frac{{c}_{1}}{{k}_{1}}$$

Equations 14 and 15 are obtained from 12 and 13 through Laplace transformation:

14 $$ F\left( s \right) = k_{1} X\left( s \right) + c_{1} \left( {sX\left( s \right) - sZ\left( s \right)} \right) $$

15 $$ sZ\left( s \right) = \frac{1}{1 + \beta }\left( {sX\left( s \right) - \frac{\alpha }{\gamma }Z\left( s \right)} \right) $$

From Eq. 15, it can be concluded that:

16 $$ Z\left( s \right) = \frac{sX\left( s \right)}{{\left( {1 + \beta } \right)s + \frac{\alpha }{\gamma }}} $$

Equation 16 is substituted into Eq. 14:

17 $$ F\left( s \right) = k_{1} X\left( s \right) + c_{1} \left( {sX\left( s \right) - \frac{{s^{2} X\left( s \right)}}{{\left( {1 + \beta } \right)s + \frac{\alpha }{\gamma }}}} \right) $$

From Eq. 17, the formula for calculating the complex stiffness can be derived:

18 $$ K_{v} \left( s \right) = \frac{F\left( s \right)}{{X\left( s \right)}} = k_{1} + c_{1} s - \frac{{c_{1} s^{2} }}{{\left( {1 + \beta } \right)s + \frac{\alpha }{\gamma }}} $$

The complex stiffness converted to the frequency domain yields the following equation:

19 $$ K_{v} \left( \omega \right) = \frac{F\left( \omega \right)}{{X\left( \omega \right)}} = k_{1} + c_{1} i\omega + \frac{{c_{1} \omega^{2} }}{{\left( {1 + \beta } \right)i\omega + \frac{\alpha }{\gamma }}} $$

By further deriving from the above equation, the amplitude of the real part and imaginary part of the response force, denoted as ${F}_{v0Re}$ and ${F}_{v0lm}$ respectively, under a sinusoidal excitation of amplitude ${x}_{0}$, can be obtained:

20 $$ F_{{v0{\text{Re}} }} = \left( {k_{1} + \frac{{c_{1} \omega^{2} \alpha \gamma }}{{\left( {1 + \beta } \right)^{2} \omega^{2} \gamma^{2} + \alpha^{2} }}} \right)x_{0} $$

21 $$ F_{v0lm} = \left( {c_{1} \omega - \frac{{c_{1} \omega^{3} \gamma^{2} \left( {1 + \beta } \right)}}{{\left( {1 + \beta } \right)^{2} \omega^{2} \gamma^{2} + \alpha^{2} }}} \right)x_{0} $$

#### New model

Because the Frequency-dependent model cannot accurately describe the viscoelastic properties of rubber, this paper proposes a Frequency-dependent model based on the High-Order Derivative Frequency-dependent model. The relationship between force and displacement in this new model is given by:

22 $$_{0} D_{t}^{\beta } F +_{0} D_{t}^{\alpha } \frac{{c_{2} }}{{c_{1} }}F + \frac{{k_{2} }}{{c_{1} }}F =_{0} D_{t}^{\beta } \left( {k_{1} + k_{2} } \right)x +_{0} D_{t}^{\alpha } \frac{{c_{2} }}{{c_{1} }}k_{1} x + \frac{{k_{2} k_{1} }}{{c_{1} }}x +_{0} D_{t}^{\alpha + \beta } c_{2} x $$

In the equation, α and β represent the order of the fractional derivatives, which range $(\text{0,1})$; $k_{1} ,k_{2} ,c_{1} ,c_{2}$ are the elastic modulus and viscosity coefficients of the model, respectively; and ${{}_{0}{}D}_{t}^{\alpha }$ is the Riemann–Liouville fractional derivative operator.

## ${{}_{0}{}D}_{t}^{\alpha }$ can be definition by Eq. 23:

23 $$_{0} D_{t}^{\alpha } f\left( t \right) \approx \frac{1}{{\Gamma \left( {n - \alpha } \right)}}\frac{{d^{n} }}{{dt^{n} }}\int_{\alpha }^{t} {\frac{f\left( x \right)}{{\left( {t - x} \right)^{\alpha - n + 1} }}dx} $$

In other words, $f(t)$ is first to do $(n-\alpha )$ fractional integration, and then take the $n$ derivative, $n$ is 1.

Based on the Frequency-dependent model, changing the damping to a sticky pot with fractional derivative of displacement can better describe the viscoelastic properties of rubber. When α and β are both 1, the new model will be equal to the Frequency-dependent model, so the mechanical properties of the new model already include the mechanical properties that the Frequency-dependent model can represent.

Equations 23 are obtained 22 through Laplace transformation:

24 $$ s^{\beta } F\left( s \right) + s^{\alpha } \frac{{c_{2} }}{{c_{1} }}F\left( s \right) + \frac{{k_{2} }}{{c_{1} }}F\left( s \right) = s^{\beta } \left( {k_{1} + k_{2} } \right)X\left( s \right) + s^{\alpha } \frac{{c_{2} k_{1} }}{{c_{1} }}X\left( s \right) + \frac{{k_{2} k_{1} }}{{c_{1} }}X\left( s \right) + s^{\alpha + \beta } c_{2} X\left( s \right) $$

From Eq. 23, the formula for calculating the complex stiffness can be derived:

25 $$ K_{v} \left( s \right) = \frac{F\left( s \right)}{{X\left( s \right)}} = \frac{{\left( {k_{1} + k_{2} } \right)\left( s \right)^{\beta } + \lambda_{1} k_{1} \left( s \right)^{\alpha } + \lambda_{2} k_{1} + c_{2} \left( s \right)^{\alpha + \beta } }}{{\left( s \right)^{\beta } + \lambda_{1} \left( s \right)^{\alpha } + \lambda_{2} }} $$

$${\lambda }_{1}= \frac{{c}_{2}}{{c}_{1}}$$; $${\lambda }_{2}= \frac{{k}_{2}}{{c}_{1}}$$。

The complex stiffness of the new model in the frequency domain can be derived as:

26 $$ K_{v} \left( \omega \right) = \frac{F\left( \omega \right)}{{X\left( \omega \right)}} = \frac{{\left( {k_{1} + k_{2} } \right)\left( {i\omega } \right)^{\beta } + \lambda_{1} k_{1} \left( {i\omega } \right)^{\alpha } + \lambda_{2} k_{1} + c_{2} \left( {i\omega } \right)^{\alpha + \beta } }}{{\left( {i\omega } \right)^{\beta } + \lambda_{1} \left( {i\omega } \right)^{\alpha } + \lambda_{2} }} $$

27 $$ (i\omega )^{\alpha } = \omega^{\alpha } e^{{{{i\pi \alpha } \mathord{\left/ {\vphantom {{i\pi \alpha } {2 + 2n\pi \alpha }}} \right. \kern-0pt} {2 + 2n\pi \alpha }}}} $$

Setting n = 0 as the principal root, the result is as follows:

28 $$ (i\omega )^{\alpha } = \omega^{\alpha } e^{{{{i\pi \alpha } \mathord{\left/ {\vphantom {{i\pi \alpha } 2}} \right. \kern-0pt} 2}}} $$

From Euler's formula, we can obtain:

29 $$ (i\omega )^{\alpha } = \omega^{\alpha } (cos(\frac{\alpha \pi }{2}) + isin(\frac{\alpha \pi }{2})) $$

Substituting Eq. 26 into Eq. 23, we have:

30 $$ K_{v} \left( \omega \right) = \frac{{\left[ \begin{gathered} \left( {k_{1} + k_{2} } \right)\omega^{\beta } \cos \left( {\frac{\beta \pi }{2}} \right) + \lambda_{1} k_{1} \omega^{\alpha } \cos \left( {\frac{\alpha \pi }{2}} \right) + \hfill \\ \lambda_{2} k_{1} + c_{2} \omega^{\alpha + \beta } \cos \left( {\frac{\alpha + \beta }{2}\pi } \right) + \hfill \\ \left( {k_{1} + k_{2} } \right)\omega^{\beta } \sin \left( {\frac{\beta \pi }{2}} \right)i + \lambda_{1} k_{1} \omega^{\alpha } \sin \left( {\frac{\alpha \pi }{2}} \right)i + \hfill \\ c_{2} \omega^{\alpha + \beta } \sin \left( {\frac{\alpha + \beta }{2}\pi } \right)i \hfill \\ \end{gathered} \right]}}{{\left[ \begin{gathered} \omega^{\beta } \cos \left( {\frac{\beta \pi }{2}} \right) + \lambda_{1} \omega^{\alpha } \cos \left( {\frac{\alpha \pi }{2}} \right) + \lambda_{2} + \hfill \\ \omega^{\beta } \sin \left( {\frac{\beta \pi }{2}} \right)i + \lambda_{1} \omega^{\alpha } \sin \left( {\frac{\alpha \pi }{2}} \right)i \hfill \\ \end{gathered} \right]}} $$

By further deriving from the above equations, the magnitudes of the real part and imaginary part of the response force, denoted as ${F}_{v0Re}$ and ${F}_{v0lm}$ respectively, under a sinusoidal excitation of amplitude ${x}_{0}$ can be obtained:

31 $$ F_{{vo{\text{Re}} }} = \frac{\begin{gathered} \left[ \begin{gathered} \left( {k_{1} + k_{2} } \right)\omega^{\beta } \cos \left( {\frac{\beta \pi }{2}} \right) + \lambda_{1} k_{1} \omega^{\alpha } \cos \left( {\frac{\alpha \pi }{2}} \right) + \hfill \\ \lambda_{2} k_{1} + c_{2} \omega^{\alpha + \beta } \cos \left( {\frac{\alpha + \beta }{2}\pi } \right) \hfill \\ \end{gathered} \right]\left[ \begin{gathered} \omega^{\beta } \cos \left( {\frac{\beta \pi }{2}} \right) + \hfill \\ \lambda_{1} \omega^{\alpha } \cos \left( {\frac{\alpha \pi }{2}} \right) + \lambda_{2} \hfill \\ \end{gathered} \right] - \hfill \\ \left[ \begin{gathered} \left( {k_{1} + k_{2} } \right)\omega^{\beta } \sin \left( {\frac{\beta \pi }{2}} \right)i + \lambda_{1} k_{1} \omega^{\alpha } \sin \left( {\frac{\alpha \pi }{2}} \right)i + \hfill \\ c_{2} \omega^{\alpha + \beta } \sin \left( {\frac{\alpha + \beta }{2}\pi } \right)i \hfill \\ \end{gathered} \right]\left[ \begin{gathered} \omega^{\beta } \sin \left( {\frac{\beta \pi }{2}} \right)i + \hfill \\ \lambda_{1} \omega^{\alpha } \sin \left( {\frac{\alpha \pi }{2}} \right)i \hfill \\ \end{gathered} \right] \hfill \\ \end{gathered} }{{\left[ \begin{gathered} \omega^{\beta } \cos \left( {\frac{\beta \pi }{2}} \right) + \hfill \\ \lambda_{1} \omega^{\alpha } \cos \left( {\frac{\alpha \pi }{2}} \right) + \lambda_{2} \hfill \\ \end{gathered} \right]^{2} + \left[ \begin{gathered} \omega^{\beta } \sin \left( {\frac{\beta \pi }{2}} \right) + \hfill \\ \lambda_{1} \omega^{\alpha } \sin \left( {\frac{\alpha \pi }{2}} \right) \hfill \\ \end{gathered} \right]^{2} }}x_{0} $$

32 $$ F_{volm} = \frac{\begin{gathered} \left[ \begin{gathered} \left( {k_{1} + k_{2} } \right)\omega^{\beta } \sin \left( {\frac{\beta \pi }{2}} \right) + \lambda_{1} k_{1} \omega^{\alpha } \sin \left( {\frac{\alpha \pi }{2}} \right) + \hfill \\ c_{2} \omega^{\alpha + \beta } \sin \left( {\frac{\alpha + \beta }{2}\pi } \right) \hfill \\ \end{gathered} \right]\left[ \begin{gathered} \omega^{\beta } \cos \left( {\frac{\beta \pi }{2}} \right) + \hfill \\ \lambda_{1} \omega^{\alpha } \cos \left( {\frac{\alpha \pi }{2}} \right) + \lambda_{2} \hfill \\ \end{gathered} \right] - \hfill \\ \left[ \begin{gathered} \left( {k_{1} + k_{2} } \right)\omega^{\beta } \cos \left( {\frac{\beta \pi }{2}} \right) + \lambda_{1} k_{1} \omega^{\alpha } \cos \left( {\frac{\alpha \pi }{2}} \right) + \hfill \\ \lambda_{2} k_{1} + c_{2} \omega^{\alpha + \beta } \cos \left( {\frac{\alpha + \beta }{2}\pi } \right) \hfill \\ \end{gathered} \right]\left[ \begin{gathered} \omega^{\beta } \sin \left( {\frac{\beta \pi }{2}} \right) + \hfill \\ \lambda_{1} \omega^{\alpha } \sin \left( {\frac{\alpha \pi }{2}} \right) \hfill \\ \end{gathered} \right] \hfill \\ \end{gathered} }{{\left[ \begin{gathered} \omega^{\beta } \cos \left( {\frac{\beta \pi }{2}} \right) + \hfill \\ \lambda_{1} \omega^{\alpha } \cos \left( {\frac{\alpha \pi }{2}} \right) + \lambda_{2} \hfill \\ \end{gathered} \right]^{2} + \left[ \begin{gathered} \omega^{\beta } \sin \left( {\frac{\beta \pi }{2}} \right) + \hfill \\ \lambda_{1} \omega^{\alpha } \sin \left( {\frac{\alpha \pi }{2}} \right) \hfill \\ \end{gathered} \right]^{2} }}x_{0} $$

The above equations will be used for the subsequent parameter identification of the viscoelastic element.

## Parameter identification of the parameterized model for rubber bushing

In the parameter identification process of the rubber bushing model, the elastic unit and friction unit are first identified using quasi-static loading data. Subsequently, the viscoelastic unit is identified by combining the dynamic stiffness data.

### Parameter identification of the elastic and friction units

**Figure 12:**
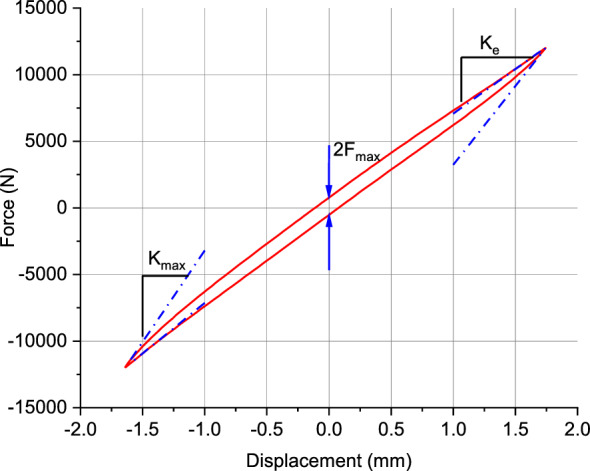
Force–displacement curve under quasi-static loading test.

Parameter identification is performed using static loading test data. The static elastic stiffness ${K}_{e}$ of the elastic unit, as shown in the Fig. 12, can be approximate by the slope of the curve near the limit position of displacement. The maximum friction force ${F}_{fmax}$ in the friction model expressed by half the vertical distance between the upper and lower limits of the hysteresis loop. The maximum slope of the curve is ${K}_{max}$.

The parameter ${x}_{2}$ in the friction unit can be determined using Eq. 32.

33 $$ x_{2} = {{F_{f\max } } \mathord{\left/ {\vphantom {{F_{f\max } } {(k_{max} - k_{e} )}}} \right. \kern-0pt} {(k_{max} - k_{e} )}} $$

**Figure 13:**
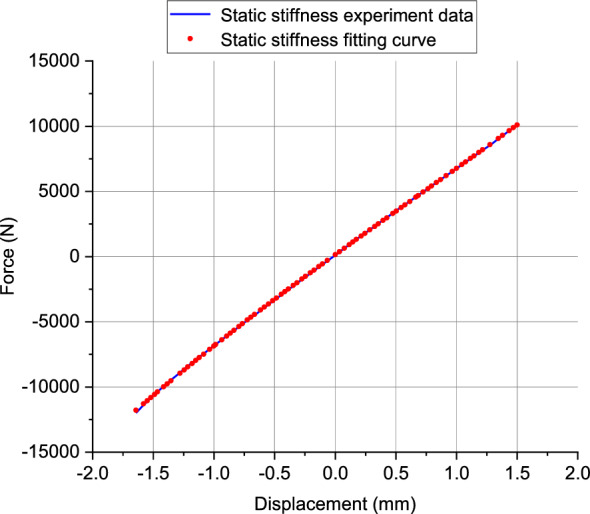
Fitting the curve of the elastic unit.

By aligning the upper and lower boundary curves of the hysteresis loop in Fig. 12 through translation, the force–displacement test curve for the elastic component is obtained. Using the data from this curve, a 3rd-degree polynomial spring model is fitted as shown in Fig. 13.

Results of parameter identification for the elastic unit and friction unit are shown in Table 4.

**Table 4:** Parameter identification results for the elastic and friction units.

Model units	Parameters	Results
Elastic unit	${K}_{e}$	6663.68
	$n$	3
	${a}_{3}$	85.12
	${a}_{2}$	-187.2
	${a}_{1}$	6782
	${a}_{0}$	647.662
Frictional unit	${F}_{fmax}/(\text{N})$	0.1257
	${x}_{2}/(\text{mm})$	0.1257

### Parameter identification of the viscoelastic unit

The parameter identification of the viscoelastic unit involves a large number of parameters, resulting in a significant computational burden. Parameter identification for the viscoelastic unit is typically performed using algorithms such as least squares^24^, genetic algorithms^25^, and particle swarm optimization.

Because the model established in this paper has many parameters and strong nonlinear. The PSO algorithm is often used to solve the optimization problem with many parameters, wide range and strong nonlinear. Therefore, this paper selects PSO algorithm for optimization. However, the PSO algorithm is prone to premature convergence, meaning it may get trapped in local optima and fail to explore the entire search space. Due to the premature convergence problem of the particle swarm optimization algorithm, where it gets trapped in a local optimal solution, genetic algorithm has the ability of mutation. The proposed improved particle algorithm is proposed based on the genetic and mutation ideas. When the particle swarm optimization algorithm gets trapped in a local optimal solution, a new particle swarm is generated by mutating it to seek a better solution and thus avoid premature convergence.

To improve the speed of optimization, a random particle is selected from the particle swarm during the velocity update process. By controlling the particle's velocity update in three directions, the speed of particle optimization is enhanced, and it helps prevent getting trapped in local optima. The velocity update equation is as follows:

34 $$ \begin{gathered} v_{t + 1} = wv_{t} + c_{1} r_{1} (p_{b} - x_{t} ) + c_{2} r_{2} (g_{b} - x_{t} ) \hfill \\ + c_{3} r_{3} (p_{s} - x_{t} ) \hfill \\ \end{gathered} $$

In the Eq. 33: ${c}_{3}$ is the learning factor; ${r}_{3}$ is a random number in the range (0,1); ${p}_{s}$ is the randomly selected particle from the current particle swarm.

To prevent getting trapped in local optima, the results of each optimization iteration are evaluated. If the historical best fitness of the particle swarm remains unchanged after the current iteration is completed, the entire particle swarm undergoes crossover and mutation operations similar to those in genetic algorithms. This generates new particles and changes the search direction, thereby avoiding local optima.

The crossover operation is performed in a real-valued encoding format. For particles that meet the crossover condition, one random particle is selected for the crossover operation. The specific method is as follows:

35 $$ \begin{gathered} x_{k} = x_{k} (1 - \sigma ) + x_{l} \hfill \\ x_{l} = x_{l} (1 - \sigma ) + x_{k} \hfill \\ \end{gathered} $$

In the Eq. 34: ${x}_{k}$ represents the particle that satisfies the crossover condition, ${x}_{l}$ is the randomly selected particle, and $\sigma$ is a random number in the range (0,1).

The mutation operation applies different mutation probabilities to different particles. Therefore, the particle swarm is sorted in ascending order based on their fitness values, where particles with higher fitness values have higher mutation probabilities. The specific method is as follows:

36 $$ P_{m} = 0.5 - 0.01\left( {{i \mathord{\left/ {\vphantom {i n}} \right. \kern-0pt} n}} \right) $$

In the Eq. 35: ${P}_{m}$ represents the mutation probability, and i represents the index of the particle in the population, ranging from 1 to n.

The mutation operation selects the j-th dimension of the i-th particle for mutation. The mutation method is as follows:

37 $$ x_{ij} = {{\left( {max(j) + min(j)} \right)} \mathord{\left/ {\vphantom {{\left( {max(j) + min(j)} \right)} 2}} \right. \kern-0pt} 2} + \left( {max(j) - min(j)} \right)\left( {r - 0.5} \right) $$

In the Eq. 36: $max(j)$ represents the upper bound of the j-th dimension of the particle, $min(j)$ represents the lower bound of the j-th dimension of the particle, and r is a random number in the range (0, 1).

Fitness function of the algorithm:

38 $$ F_{obj} = \sum\limits_{i = 1}^{n} {\left[ {\left( {\frac{{k_{dyn}^{i} - k_{dyn\_t}^{i} }}{{k_{dyn\_t}^{i} }}} \right)^{2} } \right]} $$

In the Eq. 37: $n$ represents the number of operating conditions being considered; ${k}_{dyn\_t}^{i}$ represents the experimentally measured dynamic stiffness data; ${k}_{dyn}^{i}$ represents the dynamically calculated dynamic stiffness for the i-th operating condition.

During the identification process, it is necessary to ensure that the data fitted during the model calculation does not have significant errors. Therefore, a constraint is established:

39 $$ \left| {\frac{{k_{dyn}^{i} - k_{dyn\_t}^{i} }}{{k_{dyn\_t}^{i} }}} \right| \le 0.1 $$

Calculate the dynamically calculated dynamic stiffness of the bushing using Eq. 39:

40 $$ \begin{gathered} F_{0} = \sqrt {(F_{e0} + F_{f0} + F_{{v0{\text{Re}} }} )^{2} + (F_{{v0{\text{lm}}}}^{2} )} \hfill \\ K_{dyn} = {{F_{0} } \mathord{\left/ {\vphantom {{F_{0} } {x_{0} }}} \right. \kern-0pt} {x_{0} }} \hfill \\ \end{gathered} $$

**Figure 14:**
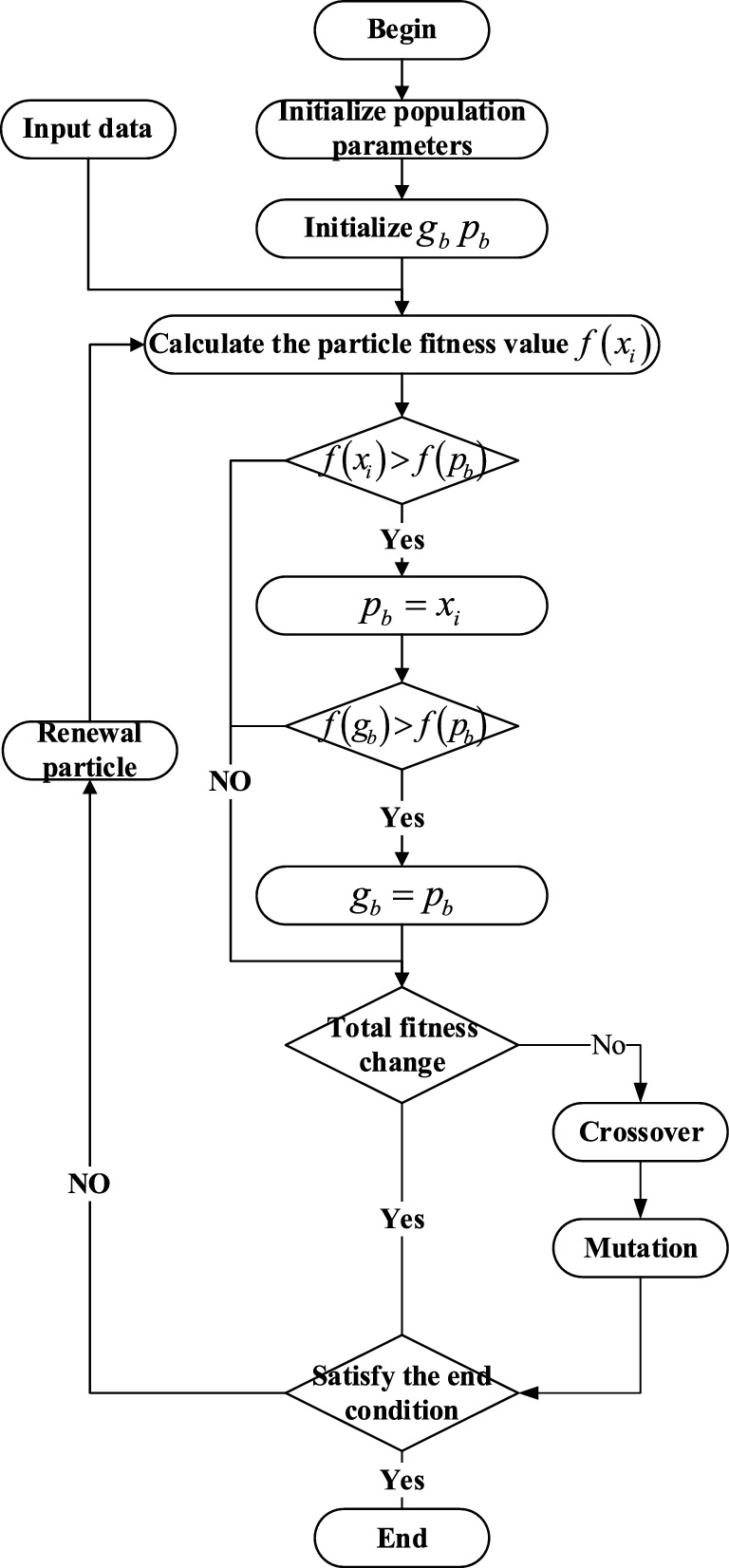
MPSO algorithm flow.

The two types of model parameters will be identified using the MPSO (Modified Particle Swarm Optimization) and PSO (Particle Swarm Optimization) algorithms separately. The MPSO algorithm will follow the process outlined in Fig. 14.

The specific parameter settings are provided in Table 5 and Table 6. Both models are selected for parameter identification using dynamic stiffness data at frequencies of 1, 10, 20, 30, 40, 50, 60, and 70 Hz, with an amplitude of 0.2 mm.

**Table 5:** MPSO and PSO parameter settings for the new model.

Algorithm	Parameters	Values
PSO	Particle dimensions	6
	n	50
	$$[{x}_{(1-4)min} , {x}_{(1-4)max}]$$	[0,500]
	$[{x}_{(4-6)min} , {x}_{(4-6)max}]$	$[\text{0,1}]$
	$w$	1.2
	${c}_{1}$	1.5
	${c}_{2}$	1.5
	$$[{v}_{(1-4)min} , {v}_{(1-4)max}]$$	[-50,50]
	$[{v}_{(4-6)min} , {v}_{(4-6)max}]$	$[-\text{1,1}]$
MPSO	${c}_{3}$	1.5
	Crossover probability	0.8

**Table 6:** MPSO and PSO Parameter Settings for the Frequency-dependent Model.

Algorithm	Parameters	Values
PSO	Particle dimensions	4
	n	50
	$$[{x}_{min} , {x}_{max}]$$	[0 ,500]
	$w$	1.2
	${c}_{1}$	1.5
	${c}_{2}$	1.5
	$$[{v}_{min} , {v}_{max}]$$	[-50 ,50]
MPSO	${c}_{3}$	1.5
	Crossover probability	0.8

**Figure 15:**
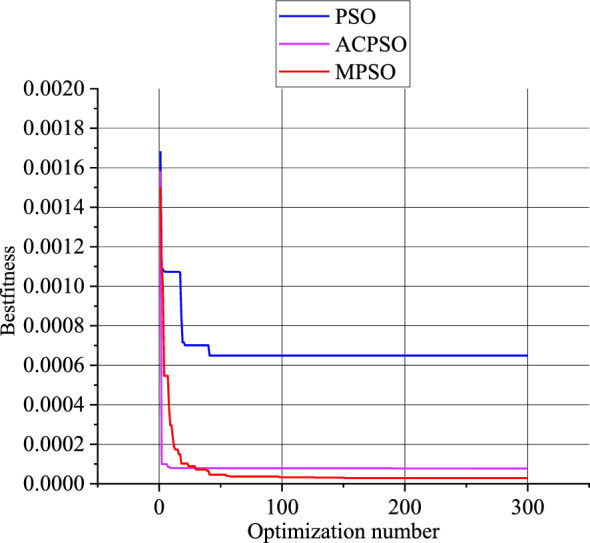
Comparison of optimization algorithms for the new model.

**Figure 16:**
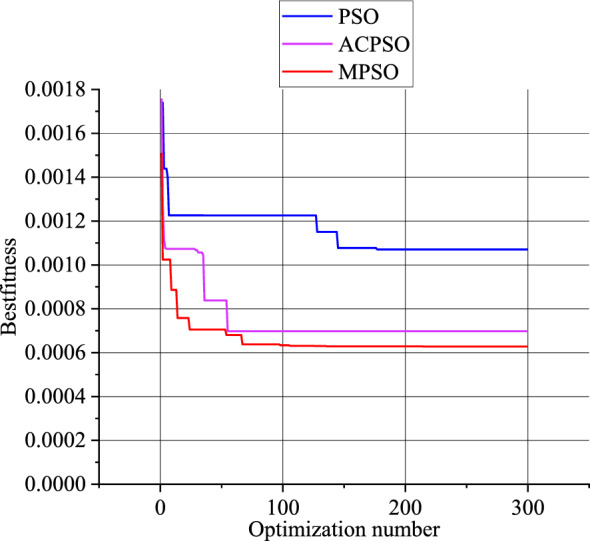
Comparison of optimization algorithms for the frequency-dependent model.

To verify the reliability of the MSPO algorithm proposed in this paper, the optimization effects of the PSO algorithm, the Adaptive chaotic particle swarm optimization (ACPSO) algorithm, and the MPSO were compared. From Fig. 15 to Fig. 16, it can be observed that both for the new model and the frequency-dependent model, the MPSO algorithm demonstrates stronger optimization capabilities compared to the PSO algorithm and the ACPSO optimization algorithm.

For the new model, the particle is represented as $x=({k}_{1},{k}_{2},{c}_{1},{c}_{2},\alpha ,\beta )$. Both MPSO and PSO algorithms will have a maximum of 300 iterations.

For the frequency-dependent model, the particle is represented as $x=({k}_{1},{k}_{2},{c}_{1},{c}_{2})$. Both MPSO and PSO algorithms will have a maximum of 300 iterations.

**Figure 17:**
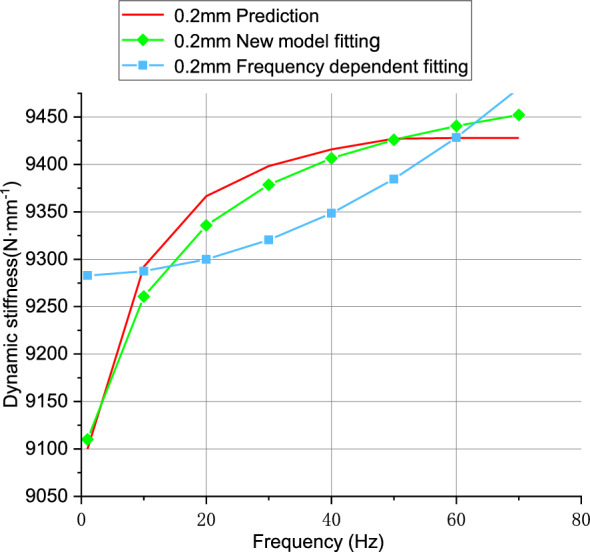
Fitting results for 0.2 mm amplitude model.

From Fig. 17, it can be observed that at a 0.2 mm amplitude, the new model exhibits better fitting performance compared to the frequency-dependent model. The error results are shown in Table 7.

**Table 7:** Model Fitting Errors.

Model	Errors (%)
New model	1.49
Frequency-dependent	5.33

The error results also indicate that the new model exhibits better fitting performance, thereby improving the model accuracy.

The identification results of unknown parameters of the two dynamic models are shown in Table 8.

**Table 8:** Identification Results of Rubber Bushing Parameters at 0.2 mm Amplitude.

Model	Parameters	Results
New model	${k}_{1}$	8.5215
	${k}_{2}$	58.5935
	${c}_{1}$	2.1749
	${c}_{2}$	14.081
	$\alpha$	0.9014
	$\beta$	0.0147
Frequency-dependent	${k}_{1}$	194.1252
	${k}_{2}$	0.1164
	${c}_{1}$	4.5061
	${c}_{2}$	259.6623

**Figure 18:**
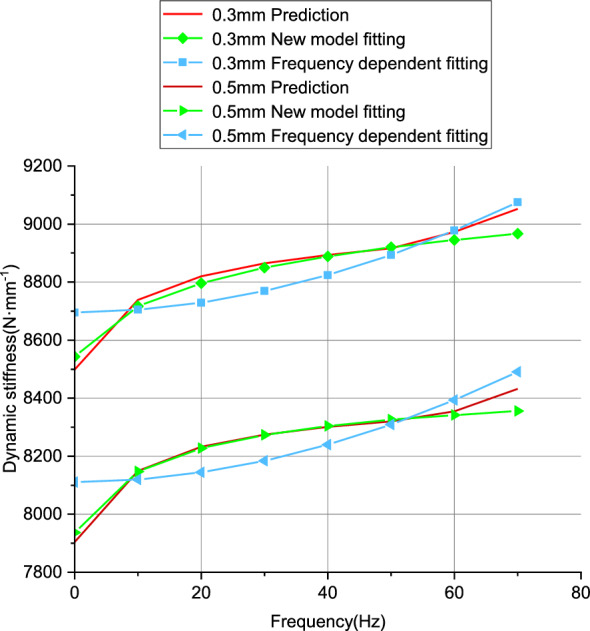
Fitting results of models for 0.3 mm and 0.5 mm data.

Both models were selected for parameter identification using dynamic stiffness data at frequencies of 1, 10, 20, 30, 40, 50, 60, and 70 Hz, at amplitudes of 0.3 mm and 0.5 mm. The results are shown in Fig. 18.

From Fig. 18, it can be observed that for both 0.3 mm and 0.5 mm amplitudes, the new model exhibits better fitting performance compared to the frequency-dependent model. The error results are shown in Table 9.

**Table 9:** Model fitting errors.

Models	Amplitudes (mm)	Errors (%)
New model	0.3	2.56
	0.5	1.67
Frequency-dependent	0.3	6.17
	0.5	7.16

The error results also indicate that the new model exhibits better fitting performance, thereby improving the model accuracy.

## Conclusion

This study conducted experiments on the rear suspension sub-frame rubber bushing of a certain electric vehicle model. The Y-direction of the rubber bushing was selected as the research object, and the PSO-BP neural network was used to predict the dynamic stiffness test data of the rubber bushing. To improve the accuracy of the bushing model, a new model was proposed based on the Frequency-dependent model when establishing the dynamic model. The parameter identification of the models and a comparison of the fitting accuracy between the two models were performed, leading to the following conclusions:

1. The proposed MPSO algorithm for parameter identification demonstrated stronger optimization capability compared to the PSO algorithm, highlighting its practical value.
2. The new model exhibited higher fitting accuracy compared to the Frequency-dependent model, indicating its practicality and usefulness.

## Data Availability

The data that support the findings of this study are available from the corresponding author upon reasonable request.

## Code availability

Codes supporting the results of this study are available upon reasonable request by the corresponding author.
